# Mechanism of Action for rTMS: A Working Hypothesis Based on Animal Studies

**DOI:** 10.3389/fphys.2017.00457

**Published:** 2017-06-30

**Authors:** Thangavelu Soundara Rajan, Maria F. M. Ghilardi, Hoau-Yan Wang, Emanuela Mazzon, Placido Bramanti, Domenico Restivo, Angelo Quartarone

**Affiliations:** ^1^Department of Experimental Neurology and Clinical Neurophysiology, Centro Neurolesi Bonino Pulejo (IRCCS)Messina, Italy; ^2^Department of Physiology, Pharmacology and Neuroscience, Sophie Davis School for Biomedical Education at City College of New York, City University of New YorkNew York, NY, United States; ^3^The Fresco Institute for Parkinson's and Movement Disorders, NYU Langone Medical CenterNew York, NY, United States; ^4^Department of Neurology, Nuovo Garibaldi HospitalCatania, Italy; ^5^Department of Biomedical, Dental Sciences and Morphological and Functional Images, University of MessinaMessina, Italy

**Keywords:** TMS, BDNF-TrKB signaling, NMDA, long-term potentiation, neuroprotection

## Abstract

Experiments in rodents have elucidated some of the molecular mechanisms underlying repetitive transcranial magnetic stimulation (rTMS). These studies may be useful in a translational perspective so that future TMS studies in rodents can closely match human TMS protocols designed for therapeutic purposes. In the present work we will review the effects of rTMS on glutamate neurotransmission which in turn induce persistent changes in synaptic activity. In particular, we will focus on the role of NMDA and non-NMDA transmission and on the permissive role of BDNF-TrKB interaction in the rTMS induced after-effects.

Experiments in rodents are indeed valuable in providing information about the molecular mechanisms of action of repetitive transcranial magnetic stimulation (rTMS). From the hitherto published work on TMS/rTMS in rodents of almost two decades, it is possible to formulate a working hypothesis on the mechanism of action of rTMS so that future TMS studies in rodents can closely match human TMS protocols. The effects of rTMS in rodents are greatly dependent on: (1) the frequency and field intensity of the stimulation; (2) the acute and chronic mode of treatment; (3) the total number of pulses; (4) the shape and dimension of coils; and (5) the state, either anesthetized or awake, of the animals. Experimental evidences in rodents indicate that rTMS produces complex neurobiochemical effects such as induction of immediate early genes, changes in modulation of neurotransmitters release, effects on glutamate AMPA receptor/NMDA receptor expression (influencing calcium ion dynamics), action on neuroendocrine systems, neuroprotective effects by reducing oxidative stress and inflammation, and a powerful activation of neurotrophic factors (Cirillo et al., 2017). These molecular effects may modify the intrinsic and extrinsic electrophysiological properties of neurons and reprogram the expression of excitatory and inhibitory neurotransmitters and their cognate receptors, which lead to long-lasting synaptic plasticity-related changes like Long-term potentiation (LTP) and depression (LTD) phenomena (Chervyakov et al., 2015; Cirillo et al., 2017).

Having said that rTMS provides sustained effects, how does rTMS induce long lasting changes in cortical excitability? An important upstream regulator of synaptic plasticity is the BDNF-TrkB system. Indeed, we showed that 5-day rTMS enhances BDNF binding affinity for TrkB, BDNF-TrkB signaling, and NMDA receptor–TrkB interaction in rat prefrontal cortex (Wang et al., 2011). Interestingly, the same rTMS protocol increases BDNF binding affinity for TrkB and enhances BDNF–TrkB signaling in the peripheral lymphocytes of both rats and humans (Wang et al., 2011). These results suggest that the long lasting excitatory effects of rTMS are, at least in part, mediated by glutamatergic NMDA interaction. Indeed, the role of NMDA transmission has been confirmed by several human TMS studies exploring the after effects of anti-glutamatergic drugs on cortical excitability and cortical plasticity (Schwenkreis et al., 2005; Wankerl et al., 2010). However, it is well known that TMS of the motor system produces high frequency repetitive discharge in the corticospinal cells with a frequency of about 700 Hz (Di Lazzaro et al., 1998). Therefore, it is unlikely that TMS may directly interact with NMDA receptors since they may work as a low-pass filter for high frequencies, due to their slow depolarizing currents (Di Lazzaro et al., 1998). Based on these considerations, it is then plausible that at least single pulse TMS may preferentially activate AMPA receptors that open quickly and briefly. This effect of TMS on AMPA receptors has been confirmed by the results of single pulse TMS studies with ketamine in humans (Di Lazzaro et al., 2003). Ketamine, an NMDA antagonist, blocks NMDA receptor activity and enhances non-NMDA transmission through an increased release of endogenous glutamate. Indeed, motor evoked responses were greatly enhanced by ketamine, suggesting that the excitatory effects of single pulse TMS are mediated by short-lasting effects on AMPA transmission (Di Lazzaro et al., 2003). High frequency rTMS-induced expression of the GLUR1 subunit of the AMPA receptors has been demonstrated in rats (Gersner et al., 2011). An important paradox is therefore to reconcile the preferential effect of TMS on AMPA receptors with the long-term excitatory effects that should be mediated by the activity of NMDA receptors. When membrane potential is at rest, NMDA receptors are blocked by Mg^++^ ions. Once sufficient numbers of AMPA receptors are activated, the membrane is depolarized from resting to active potential, the magnesium blockade is relieved, and the NMDA receptors are opened (Fleming and England, 2010). Therefore, it is possible that when TMS is applied in a repetitive fashion AMPA receptors are recruited in a sufficient number to trigger NMDA transmission. The opening of NMDA receptors increases calcium influx and activates several calcium-sensitive signaling pathways (including phosphorylation of existing AMPA receptors and synthesizing new AMPA receptors) that produce long-term changes in both the presynaptic and postsynaptic neurons that ultimately leads to increased synaptic strength (Malenka and Bear, 2004). Long-term potentiation phenomena are empowered, at least in part, by retrograde signals that further release glutamate and BDNF. Therefore, we postulate that the TMS modulation of BDNF-TrkB pathway could play a permissive role in determining the NMDA dependent after-effects on synaptic plasticity (Wang et al., 2011). This complicate molecular machinery may explain the slow building up of rTMS-induced after-effects (usually 5–10 min) in humans (Quartarone et al., 2006; Ziemann et al., 2008).

Despite the role of post-synaptic NMDA transmission on the long lasting effects of rTMS seems very clear, on the other hand there are evidences suggesting the involvement of presynaptic glutamatergic mechanisms (Banerjee et al., 2017). Indeed, it has been demonstrated in an *in vitro* model that rTMS may increase the steady state current in the presynaptic compartment independently from NMDA postsynaptic transmission. In this way, it is likely that longer duration rTMS protocol may enhance these presynaptic steady state currents thus prolonging the TMS induced after-effects (Banerjee et al., 2017). An important consideration, which might be useful for future clinical application, is whether rTMS targets late LTP and promotes structural plasticity. In a recent paper in mouse entorhino-hippocampal slice cultures, it has been demonstrated that high-frequency (10 Hz) induces a long-lasting increase in glutamatergic synaptic strength that is accompanied by structural remodeling of dendritic spines (Vlachos et al., 2012). This possibility opens a new scenario on the therapeutic effects of rTMS even if need to be better addressed in future studies.

## Author contributions

TS: study conception, drafting of the ms; MG, HW, and PB: critical revision; EM: critical revision, analysis of animal literature; DR: critical revision, clinical implications; AQ: study conception, drafting of the ms, crafting of the ms.

### Conflict of interest statement

The authors declare that the research was conducted in the absence of any commercial or financial relationships that could be construed as a potential conflict of interest. The reviewer DP and handling Editor declared their shared affiliation, and the handling Editor states that the process nevertheless met the standards of a fair and objective review.
